# Prevalence of burnout syndrome among physiotherapist working in intensive care units and step down unit

**DOI:** 10.1186/2197-425X-3-S1-A722

**Published:** 2015-10-01

**Authors:** KT Timenetsky, CA Castro, RC Eid, D Carnieli-Cazati

**Affiliations:** Hospital Albert Einstein, Pacientes Graves, São Paulo, Brazil

## Introduction

Burnout Syndrome is defined as a condition of mental suffering work-related and associated with physiological changes resulting from stress. The burnout syndrome is experienced mainly by health professionals involved in care activities, where the greatest demand for this type of service is to deal with the emotional needs of the other, under stress.

## Objectives

Check the prevalence of burnout syndrome among health professionals working in intensive care units and step down unit.

## Methods

We applied a specific questionnaire to the physiotherapy team in the intensive care unit and step down, with questions describing the socio-demographic and occupational variables of the participants, plus specific information to describe the burnout using the Maslach Burnout Inventory (MBI). This instrument consists of 22 items, ranging from “0” as “never” to “6” as “every day”, distributed in three blocks - emotional exhaustion, depersonalization, and job satisfaction. It was used as reference values, the points established in studies conducted by GEPEB - Group of Studies and Research on Stress and Burnout. Considering as having burnout syndrome a person with high scores on Emotional Exhaustion and Depersonalization, associated with low points in Personal Achievement.

## Results

Sixty physiotherapists answered the questionnaire, the results revealed a prevalence of 11%, with the main risk factors related to workload represented as higher daily work of 12 hours or more and presence of chronic pain mainly in spine, legs and head. However when evaluated the three items separately, 58% of the physiotherapy team presents or high level of emotional exhaustion, or high level depersonalization or low level in job satisfaction.

## Conclusions

It was possible to observe low prevalence of the syndrome of burnout among professional physiotherapy, however a high risk for the development of it.
